# 

**Published:** 1905-01-15

**Authors:** 


### SUBJECT INDEX TO VOLUME LIX.

### EVENT AND COMMENT.

Advertising Dentists, a Menace or Necessity, 220.

Age Limit of Dental Practitioner, 110.

Army Dentists, 544.

Bust of Dr. J. Taft, 163.

California Dental Law Constitutional, 222.

Can Dental Operations be Made More Attractive to Patients? 379.

Cement Problem, 541.

College Course, Length of, 165.

College Fees Raised, 328.

Correspondence Schools, 112

Dealers' Exhibit Convention, 8.

Dental Journalism, 329.

Dental Literature, 115.

Dental Society Work, 325.

Dentists Eat too Much, 109.

Dignifying our Profession, 271.

Editorial Qualities, 488.

Elimination of Science Studies from the Curriculum, 57.

Emperor William's Dentist Suicides, 6.

Enthusiasm, 166.

Extracting for Regulating, 164.

Fees, 113.

Fellowship Medal, 329.

First Molar, 543.

Former Ideals Contrasted, 380.

Germany and the American Degree, 2.

Great Clinic, 273.

Hand Excavator Passing, 217.

Humbuggery, 596.

Identification, 57.

Illinois State Society Reorganization, 5.

Institute of Dental Pedagogics, 4.

Legislation, New Features, 542.

McKellops Library, 55.

Metal versus Vulcanite Plate, 164.

Miller, Prof. W. D., honored, 110.

National Association of Dental Examiners' Annual Meeting, 635.

National Association of Examiners, Has it Power? 219.

National Association of Dental Faculties' Annual Meeting, 437.

National Dental Association, Annual Meeting, 433.

New College Year.

New Cover, 60.

Nineteen Hundred and Five, 1.

Occlusion, Its Value in Prothodontia, 273.

Professional Emancipation, 58.

Prophylaxis, objections to, 168.

Protest by Berlin American Dentists, 115.

Pyorrhea Alveolaris and Prophylaxis, 491.

Qualifications for Practice in England, 545.

Quinine for Pyorrhea, 6.

Recognition of the Home Trained dentist, 381.

Reciprocal Relations, 56.

Shall we Graduate on the Time or Educational Basis? 382.

State Board Officers, Nominating, 219.

State Dental Laws, 61.

Temporary Licences abandoned, 221.

The Strained Editorial, 487.

The Year's Work, 596.

Toothpicks Banished, 595.

To Whom the Good? 326.

Tri-State Meeting, 3.

Wisconsin State Examining Board Annual Report, 116.

Young, Dr. J. L., Banquetted, 274.

### ADDRESSES AND PAPERS.

Address, President A. L. LeGro, 296.

Alveolar Abscess, Treatment of Some Phases, G. W. Cook, 285.

Amalgams and their Peculiarities, M. L. Ward, 331.

Amalgam as a Filling Material, George Zederbaum, 599.

Amalgams, Do Modern Alloys Shrink? M. L. Ward, 276.

Antiseptic Value of Chloretone, T. A. Gormley, 580.

Benefits of Post Graduate Courses, A. C. Runyan, 399.

Chemistry, its Value in Dentistry, Geo. M. Heath, 178.

Cleaning Teeth, N. S. Hoff, 233.

Cocain Pressure Anesthesia, Its Dangers and Failures, E. T. Loeffler, 546.

Discussion of Papers on Filling Materials, 613.

French Requirments and the D. D. S. in France, Chas. J. Gray, 9.

Gold as a Filling Material, J. L. Sweetnam, 609.

How much Orthodontia should the General Practitioner Do? Martin Dewey, 394.

Importance of Occlusion in Crown and Bridge Work, R. B. Howell, 554.

Life of an Army Dentist, S. W. Hussey, 576.

Nitrous Oxide Gas, Advantages and Proper Method of Administration, S. Straith, 448.

Operative Technics, Importance of Systematic Training, F. W. Gethrow, 439.

Our Income as an Element in Dental Practice, C. J. Cigrand, 347.

Painless Removal of Pulp by Chloretone, D. W. Babcock, 70.

Porcelain as a filling Material, H. C. Raymond, 604.

Porcelain Crown, a New One, C. H. Worboys, 298.

Pulp Canal Filling, Comments on, N. S. Hoff, 172.

Pulp Canals, How do you Fill them? A. W. Harlan, 171.

Pyorrhea Alveoraris, C. A. Burbridge, 503.

Reactions from Pressure Anesthesia with Cocain, E. T. Loeffler, 62.

Replantation, Transplantation and Implantation, J. F. Austin, 290.

Sanitary Artificial Dentures, N. S. Hoff, 118.

Some Changes in Dental Practice made Necessary by Modern Science, C. M. Wright, 383.

Somnoform and Its Administration, 16.

Somnoform, Reasons for Preferring it to Gas, G. C. Bowles, 460.

The Need of Oral Prophylaxis and some Results Obtained by it, E. B. Spalding, 492.

The People vs. The Dentist, Geo. Zederbaum, 224.

**REPRINTS.**

Alveolar Abscess Treatment, J. P. Buckley, 139.

Anesthesia, S. Sauvez.

Army Dental Corps, Dr. Jno. S. Marshall, 37.

Artificial Dentures, Record of 49 Cases Swallowed, A. D. Black, 304.

Cement Problem, G. C. Poundstone, 471.

Chloretone and Cocain for Extracting, R. J. Winn, 96.

Degeneracy, E. S. Talbot, 196.

Dental Caries, Some Facts, A. Lohman and E. C. Kirk, 42.

Dental Poverty, G. S. Junkerman, 29.

Desensitizing the Pulp, by Way of the Tubuli, J. E. Line, 407.

Errors of Diet, R. A. Gunn, M. D., 134.

Fractures of the Jaws, T. L. Gilmer, 195.

Gold Inlays, E. H. Allen, 247.

Liberation or Extraction of Empacted teeth, M. H. Cryer, 193.

Low Carat Solder, Why use it? W. H. Truman, 242.

Practical Treatment of Pyorrhea Alveolaris, A. F. James, 404.

Pressure Anesthesia, J. V. Conzette, 186.

Pressure Anesthesia, Difficulties, J. B. Pherrin, 190.

Professional Status, Comment on, H. C. Sexton, 92.

Prophylaxis of the Teeth, D. D. Smith, 71.

Prophylaxis and Pyorrhea Alveolaris, C. M. Wright, 300.

Public Appreciation of the Dental Profession, J. E. Nyman, 88.

Pulp Extirpation, E. MaWhinny, 308.

Somnoform, Its Physiologic Action and Administration, F. Auguilar, 19.

Tests on the Inlay Cement Problem, Joseph Head, 564.

Water Drinking, 142.

Your Breath, R. B. Tuller. 84.

INDENTS.

Abstainer and Life Insurance, 207.

Accidents, 634.

Accident, A Peculiar one, 101.

Acetezone in Septic Cellulitis, 209.

Acidity from Food, 102.

Acid or Alkaline Saliva, 104.

Agreeable Toothache Cure, 533.

Amalgam, 631.

Amalgam Crown, 212.

Amalgam, Table of Shrinkage, 283.

Analgesia, Local, 151.

Ancient and Modern Dentistry, 319.

Anti-Neuralgic Remedy, 101.

Antiseptic Root Filling, 314.

Argorol, 49.

Arranging Artificial Teeth, 483.

Arsenic, Pain from, 369.

Aseptic Canal Filling, 268.

Bandless Porcelain Crown, 205.

Barred Out, 315.

Blind Abscess, 627.

Blue Light as an Anesthetic, 53.

Bridge Repair, 266, 366.

Broach Holder Sterilizable, 416.

Burns from Acid, 481.

Button Your Cement, 263.

Calcium Sulphite, 369.

Camphor as an Antiseptic, 206.

Cardiac Syncope from Somnoform, 151.

Care of the Teeth, 371.

Caries of the Teeth, 105.

Carries, Reasons for Susceptibility 103.

Cautions Against the Use of Adrenalin, 627.

Cement and Broken Porcelain, 583.

Cleaning Hypodermic Needles, 203.

Cleaning Teeth, 102.

Cocain Solutions, 628

Coloring Impressions, 365.

Copper Sulphate for Pyorrhea, 482.

Cotton Holder, 416.

Cover for Bracket Table, 627.

Cresylone, 161.

Culter Mountain Mystery Solved, 366.

Cut Rate Drugs, 210.

Damage Suit, 214 and 431.

Death from Tooth Infection, 368.

Deciduous Dentition Defective, 367.

Dental Laws, 317.

Dentalone, 107.

Dentist a Mechanic, 204.

Dentist not Responsible, 204.

Dessicating Root Canals, 261.

Devitalized Pulps Hardened, 368.

Diagnostic Mistakes, 215.

Difficulties in Sterilizing Dental Instruments, 630.

Dishonest Practices, 629.

Drilling Sore Teeth, 262.

Dry Treatment of Burns, 263.

Dummies, 49.

Edentulous Jaws, Pecularities of, 370.

Epithelioma from Dental Operation, 103.

Ether versus Chloroform, 208.

Ethyl Chloride, 203.

Eucain and Adrenalin, 202.

Fatalities, 532.

Fatalities, 581.

Fermentable Foods, 102.

German Silver to Clean and Polish, 264.

Gold and Platinum Alloy, 628.

Gold Fillings, Anchoring, 371.

Greesed Teeth Immune, 104.

Gutta Percha for Setting Bridge, 368.

Haigs Mixed Diet, 372.

Hair Ball in the Stomach, 206.

Igorrote longs for Gold Teeth, 209.

Illinois Dental Law, 374.

Immediate Root Filling, 370.

Impressions of Cavaties, 314.

Infected Instruments, 633.

Infection from Impacted Root, 50.

Infection of the Mouth, 205.

Inlay Cementing Surface, 315

Instrument Hones, 267.

Interdental Splint, 587.

Investment Material, 210.

Investment Material, 581.

Iodine and Chinosol Contra-indicated, 265.

Judgment in Constructing Bridge Work, 104.

Less Advice, More Cash, 582.

Lewis and Clark Dental Congress, 586.

Ligature Wire, 262.

Lip Pinned, 264.

Living to Ones Self, 372.

Local Anesthetic, Reliable Method 316.

Lower Artificial Teeth, 368.

Lower Plate, 370.

Low Fusing Alloy, 202.

Malaria and Grip Cold, 101.

Manipulation of Plaster in Vulcanite Work, 628.

Matrix Burnisher of Glass, 214.

Method of Administering Somnoform, 51.

Milk, Mechanical Purification of, 211.

Millions in Gold Fillings, 424.

Moustache Holder, 262.

Mouth Wash, 101.

Mummifying Paste, 267.

Mummifying Pulp Remnants, 315.

Napkin and Cotton Roll Combination, 269.

National Meetings of 1905, 535.

New Books of 1904, 207.

New Dental College, 262.

New Property of Aluminum, 632

Nitrous Oxide and Gasoline, 102.

Nose Bleed, 533.

Number of Graduates for 1905 from the Dental Colleges, 430.

Obscene Dentistry, 365.

Obtundent, 367.

Obtunding Dentine, 203.

Occlusal Contact in Porcelain Inlays, 373.

Office Visitor, 428.

Ohio State Board of Examiners, 265.

Open Faced Crowns, 318.

Oral Hygiene, 369.

Oxygen in Surgical Infection, 212.

Palatal Defects Transmitted, 107.

Peroxide of Hydrogen, 418.

Phenol Sulforic Acid, 261.

Pink Rubber Gum, 482.

Plate, Remarkable Fit, 213.

Pneumonia, Mortality, Greatest in Flats, 204.

Poison Ivy, 481.

Polishing Crowns, 264.

Porcelain Face Incisor Crown, 266.

Porcelain Fusing, 269.

Porcelain Inlays, 374.

Porcelain versus Gold, 203.

Porcelain Working, 159.

Practical Things that Count, 322.

Precipitating Gold from other Metals, 263.

Prizes offered by N. Y. Institute of Stomatology, 484.

Professional Men, Increase of, 365.

Prophylaxis and Technics, 375.

Prophylaxis of Mercurial Stomatitis, 632.

Protection of Wounds with Zinc Chlorid, 629

Pulp Extirpation, 584.

Pulpless Sore Teeth, 365.

Pulp Mummification, 208.

Pyorrhea Alveolaris, 533.

Pyorrhea Remedies, 483.

Pyorrhea Teatment, 373, 423 and 481.

Pyrogenic Membrane, 261.

Quick Investment Material, 366.

Ratio of Sexes Treated, 52.

Recommending Nostrums, 533.

Remodeling Vulcanite Denture, 155.

Removal of Vulcanite from Porcelain Teeth, 628.

Removing Regulating Bands, 481.

Replacing a Bridge Tooth, 105.

Retaining Dentures, 202.

Retaining Loose Teeth, 419.

Return from Phillippines, 106.

Root Filling Material, 204.

Rubber Dam Retention, 367.

Rusty Instruments, 202.

Semi-lunar Ganglia Excised for Neuralgia, 205.

Separating Medium, 261.

Shading Facings for Gold Bridges, 406.

Sharpening Gates-Glidden Drills, 107.

Sodium Chloride for Pyorrhea, 418.

Sodium Dioxide, 261.

Splinting Loose Teeth, 154.

Splint for Pyorrhea Teeth, 106.

Sterilizing Fluid for Instruments, 365.

Sterilizing Instruments, 264 and 421.

Stovain, 153.

Teeth, How to Keep, 156.

Tempering, 426 and 482.

Temporary Dressing, 261.

The Best Course of Study for a Dentist, 631.

The Prosthodontist, 589.

Therapeutics, Downfall of, 158.

Tin, 630.

To Groove Gold Inlay, to Attach Cement, 627.

To Keep a Gingival Margin Cavity Dry without Rubber Dam, 629.

Toothache, 101 and 366.

Tooth Bleaching, 105.

Tooth Brush, 417.

Treatment of Pyorrhea Alveolaris, 629.

Tri-Chloracetic Acid, 265.

Tri-Facial Neuralgia, 367.

Vacation Necessary, 420.

Value of Association, 534.

Vulcanizer Explodes, 102.

Vulcanization Points, 150.

Water Sterilization, 263.

Wearing or Rusting Out, 482.

Whats in a Name, 536.

What the Prophylaxis Treatment Is, 633.

Why Round Faced Pluggers Should be Used, 422.

Wire Rubber Dam Ligatures, 103.

Zinc Chloride as a Protective of Wounds, 206.

ANNOUNCEMENTS.

American Dental Society of Europe, 148.

American Graduate Locates in Germany, 636.

American Society of Orthodontists, 312, 431 and 486, 593.

A New Examiner for Michigan, 637.

Appointment of Ohio State Board of Examiners, 432.

Central Michigan Dental Society, 146 and 252.

Clinics of Fraternal Dental Society 484.

Deaths of Michigan Dentists, 635.

Deaths of Ohio Dentists, 635

Detroit Dental Society Clinic, 145, 591.

Dr. H. M. Bridgeman, 636.

Eastern Indiana Dental Society, 199.

Fraternal Dental Society Officers, 46, 594.

Illinois State Dental Society, 362.

Indiana Board of Examiners, 636.

Indiana State Dental Society, 250 and 309.

Institute of Pedagogics, 594, 637.

International Dental Federation, 258.

Interstate Dental Federation, 313.

Jenkins Dental Society, 540.

Kansas State Dental Association, 146 and 200.

Kentucky State Dental Society, 46, 99, 147 and 199.

Lewis & Clark Dental Congress, 148 and 198.

Married, 635.

Michigan Board of Dental Examiners, 146 and 198.

Micihigan State Dental Association, 250, 310 and 432.

Missouri State Dental Association, 200 and 362.

National Dental Society, 99, and 251.

National Dental Societies, 309.

National Association of Dental Faculties, 146 and 199.

National Association of Examiners, 200 and 254.

New Deans, 361.

New Dental Journal, 362.

New Jersey State Dental Society,

New York Seventh District Society, 148.

252, 254 362 and 540.

Northern Indiana Dental Society, 431, 486 and 592.

Northern Ohio Dental Society, 252.

Odontological Society of Western Pennsylvania, 255.

Ohio State Board of Examiners, 251, 313 and 539.

Ohio State Dental Society, 45 and 539, 591.

Old Things, Exhibit of, 255.

Pennsylvania Examining Board, 145.

Pennsylvania State Dental Society, 309.

Seventh and Eighth District Societies of N. Y., 539.

South Dakota Board of Examiners, 199, 636.

Southern Branch National Dental Association, 45, 98 and 320.

Southern California Dental Association, 539.

Southern Indiana Dental Association, 255.

Southwestern Michigan Dental Society, 99 and 147.

State Board Journal, 592.

Stomatology Section, A. M. A., 256 and 312.

Tri-State Meeting, 147.

University of Michigan Alumni Clinic, 145.

Visiting List, 635.

West Virginia Board of Examiners, 199.

Wisconsin Board of Dental Examiners, 312.

Wisconsin State Dental Society, 310.

OBITUARY.

Andrews, W. L., 149.

Clayton, J. R., 149.

Devol, J. T., 539.

Downing, H. A., 368.

Goddard, C. L., 313.

Hudson, P. Hurd, 593.

Irwin James, T., 200.

Leslie, James, 200.

McFall, A. C., 480.

Shehan, Michael, 431.

Starr, E. T., 99.

Taft, Wm., 479.

Teague, H. P., 415.

Young, Elery C., 364.

BIBLIOGRAPHY.

College Songs, 148.

Crown and Bridge Work, Evans, 538.

Grays Anatomy, 363.

Mechanical Dentistry Manual, Warren, 258.

Notes on Dental Porcelain, Gilbert, 363.

Oral Pathology and Therapeutics, MaWhinny, 259.

Orthodontia and Orthopedia of the Face, Jackson, 46.

Therapeutics and Materia Medica Manual, 540.

Transaction of the Fourth International Dental Congress, 486, 593.

AUTOGRAPHIC.

Allen, E. H., 211, 247 and 418.

Ambler, H. L., 372.

Ames, W. V. B. 584.

Almon, H., 261.

Auguilar, F., 19, 28 and 203.

Austin, J. F., 290 and 296.

Babcock, D. W., 70 and 103.

Bannister, B. 267.

Bennett, G. A., 600.

Black, A., D., 262 and 304

Black, G. V., 600, 631.

Bowles, G. C., 460, 470 and 523.

Brewster, R., 267.

Broomell, J. N., 306 and 370.

Brophy, T. W., 418, 631.

Brown, G. H., 452.

Brown, J. H., 213.

Brush, F. C., 324.

Bulkley, J. P., 139 and 265.

Burbridge, C. A., 503, 516, 517 and 528.

Burke, G. F., 591.

Busch, W. A., 202.

Byram, J. Q., 517, 561, 619.

Campbell, Dr. 516.

Carlton, H. P., 432.

Cattell, D. M., 423.

Cheesman, F. E., 373.

Cigrand, C. J., 347 and 360.

Clapp, Geo. W., 369.

Cochran, B. L., 204.

Codding, F. H., 357.

Collins, T. J., 621.

Conzette, J. V., 186.

Cook, Geo. W., 285, 294 and 359.

Crain, E. A., M. D., 209.

Cryer, M. H., 193.

Darby, E. T., 216.

Davis, W. C., 316.

Dewey, Martin, 395.

Dufour, J., 151.

Ebersole, W. G., 53.

Fernandez, Dr. 108.

Findlayson, W. T., 628.

Forsy, Dr., 154.

Fowler, S. M., 295 and 358.

Gabriel, W. M., 202.

Garman, J. A., 45.

Gautz, Dr., 102.

Gaylord, Dr., 50.

Gethrow, F. W., 439.

Gilmer, T. L., 195 and 261.

Gormly, T. A., 580.

Goslee, H. J., 104 add 368.

Gray, Chas., J., 9.

Griffiths, Dr., 27.

Gunn, R. A., M.D., 134.

Hadley, C. J., 264.

Halstead, A. E., 205.

Hampton, C. G., 456.

Harlan, A. W., 171.

Harper, Jno. G., 205.

Hart, J. S., 368.

Haskells, Dr., 368, 370 and 371.

Head, Joseph, 564.

Heath, Geo. M., 178.

Henshaw, F. R., 534, 629.

Hershey, G. S., 627.

Heyke, J. E., 151.

Hirschfield, Wm., 105.

Hoff, N. S., 118, 172, 233, 403, 454, 520, 563 and 614

Honey, E. A., 358, 402 and 520.

Howell, R. B., 554, 563.

Hunt, G. E. 312.

Hurd, Judson, 593.

Hussey, S. W., 574.

Jackson, W. H., 617.

James, A. F., 404.

Jenkins, N. S., 161.

Johnson, C. N., 372.

Johnston, T. E., 156.

Junkerman, G. S., 29.

Keehn, C. G., 535.

Kirk, E. C., 42 and 312.

Knowles, Dr., 213.

Kraemer, C. W., 203.

Lasada, Dr., 26 and 28.

Le Gro, A. L., 296 and 467.

Line, J. E., 407.

Littlefield, John, 593.

Loffler, E. T., 62, 468 and 526, 546.

Lohman, Aug., 42.

Long, A. M., 616.

Lucas, R. C., 107.

Luckey, Benj., 369.

McCallie, T. O., 267.

McElhinney, M. G., 628.

McElroy, C. M., 591.

McInnis, F. J., 263.

McKinney, W. B., 516.

Marey, C. L., 209.

Marshall, Jno. S., 37, 600.

Mathews, Dr., 27.

MaWhinny, E., 308 and 424.

Miller, G. A., 102.

Miller, H. B., 426.

Miller, W. D., 102, 103, 104 and 105, 591.

Mitchell, L. J., 374.

Mitchell, Wm., 103.

Moody, J. D., 374.

Noyes, F. B., 585.

Nyman, J. E., 88.

Oakman, C. H., 622.

Oliver, R. T., 106.

Ottalenqui, Dr. 633.

Parmenter, G. A., 614.

Patterson, J. D., 534 and 536.

Perry, S. G., 214.

Pherrin, J. B., 190.

Platt, F. L., 377.

Poundstone, G. C., 471.

Price, W. A., 34.

Prinz, H., 432.

Ratliff, R. M., 315.

Raymond, H. C., 604 and 625.

Reaben, W. H., 627.

Reed, E. D., 420.

Rhein, M. L., 265 and 268.

Rix, T. G., 296, 358 and 402.

Rohland, Dr., 417.

Rowley, A. B., 295, 359 and 401.

Runyan, A. C., 360 and 399.

Sauvez, E., 125.

Sauerbrun, L. T., 593.

Scott, W. W., 401.

Sexton, H. C., 92.

Shattuck, W. G., 519, 623.

Sheridan, Thomas, 627.

Shores, F. W., 312.

Smith, D. D., 71, 633.

Smith, G. F., 523.

Spach, A. B., 367 and 373.

Spalding, E. B., 492 and 528.

Sparks, R. E., 262, 266 and 629.

Stephan, F. W., 481.

Straith, S., 448, 456 and 469.

Subirana, Dr., 26.

Sweetnam, J. L., 609 and 624.

Taft, J., 600.

Taggart, W. H., 314.

Talbot, E. D., 196.

Thompson, J. N., 360.

Tracy, W. D., 203.

Truman, W. H., 242, 319 and 582.

Tuller, R. B., 84.

Voyles, S. H., , 417, 422 and 630.

Wallace, J. Sim., 104.

Ward, M. L., 267, 331 and 618.

Watling, J. A., 622.

Watson, M. T., 525, 625.

Waye, E. J., 260.

White, Gordon, 106.

White, S. M., 401.

Wilbert, M. I., 627.

Williams J. Leon, 314 and 365.

Winn, R. J., 96.

Wood, C. P., 518 and 526.

Worboys, C. H., 298, 402, 527, 562 and 624.

Worthly, F. G., 533.

Wright, C. M., 300 and 383.

Younger, Dr., 155.

Zederbaum, 224s 297 and 599.

### ANNOUNCEMENTS.

The eighth annual meeting of the Southern branch of the National Dental Association will be held February 21-23, 1905, at Memphis, Tenn.

J. A. GORMAN, *Cor. Sec.*
Asheville, N. C.

OFFICERS Ohio State Dental Society; S. D. Ruggles, President, Portsmouth,; H. L. Ambler, First Vice-President, Cleveland; H. C. Brown, Second Vice-President, Columbus; F. R. Chapman, Secretary, Schultz Building, Columbus; C. I. Keely, Treasurer, Hamilton.

OFFICERS Fraternal Dental Society of St. Louis, Burton L Thorpe, President; E. P. Dameron, Vice-President; S. H. Voyles, Secretary, 3201 Washington Av.; W. E. Brown, Treasurer.

KENTUCKY STATE DENTAL SOCIETY MEETING.—The annual meeting this year will be held May 15th and 16th, at Lexington. It is intended that this shall be one of the best meetings this society has ever held, and a cordial invitation is extended to all dentists to attend. Any one having a matter in the form of a paper or clinic to present will please communicate with the Secretary at as early a date as possible.

W. M. RANDALL,
Masonic Building, Louisville, Ky.

